# Regulation of neutrophil extracellular trap formation by c-di-GMP in *Pseudomonas aeruginosa*-induced pleural infection

**DOI:** 10.3389/fimmu.2025.1610266

**Published:** 2025-12-15

**Authors:** Cao Qing, WenHui Huang, Lilu Shi, Jing Luo, Shaochu Zheng, Jinliang Kong, Ke Wang

**Affiliations:** 1 Department of Pulmonary and Critical Care Medicine, The First Affiliated Hospital of Guangxi Medical University, Nanning, Guangxi, China; 2 Department of Respiratory and Critical Care Medicine, Beijing Shijitan Hospital, Capital Medical University, Beijing, China

**Keywords:** *Pseudomonas aeruginosa*, cyclic di-GMP, pleural infection, neutrophil extracellular traps, transcriptomics

## Abstract

**Background:**

*Pseudomonas aeruginosa(P.aeruginosa)*employs c-di-GMP to govern biofilm formation and virulence, enhancing bacterial adhesion and immune evasion. In pleural infections, c-di-GMP simultaneously promotes invasion and modulates neutrophil extracellular traps (NETs), though its mechanistic role remains undefined. While c-di-GMP’s pathogenic contributions are established, its specific regulation of NETs formation during pleural infection has not been explored. Our study investigates c-di-GMP-driven NETs generation in murine infection models.

**Method:**

C57BL/6J mice were infected with *P.aeruginosa* strains expressing high (PAO1△wspF), wild-type (PAO1), or low (PAO1/plac-yhjH) c-di-GMP levels to establish pleural infection. *In vitro*, c-di-GMP levels and biofilm formation of strains were verified using pcdrA-gfp reporter, crystal violet staining, and confocal microscopy. *In vivo*, pleural histopathology was observed via HE staining, confocal microscopy and scanning electron microscopy (SEM); RNA sequencing (RNA-seq) of mouse pleural lavage fluid cells was performed, followed by Mfuzz clustering, KEGG/GO enrichment analysis, and protein-protein interaction (PPI) network construction to identify key regulatory genes. Candidate genes (e.g., *Cybb*, *Mapk14*, *Pik3cd*) were validated by qRT-PCR. NETs formation was quantified via immunofluorescence, and measurements of cell-free DNA (cf-DNA) and myeloperoxidase-DNA (MPO-DNA) complexes in pleural lavage fluid.

**Result:**

*In vitro*, PAO1△wspF showed the highest c-di-GMP level and biofilm formation, followed by PAO1 and PAO1/plac-yhjH. *In vivo*, infected mice exhibited thoracic purulent secretions and parietal pleura thickening (vs. PBS control), with no obvious morphological differences among the three infected groups. RNA-seq and Mfuzz clustering identified 5 continuously downregulated gene clusters in infected mice; KEGG enrichment showed 48 genes enriched in the ‘neutrophil extracellular trap formation’ pathway. PPI network analysis screened 10 hub genes (including *Cybb*, *Mapk14*, *Pik3cd*), whose differential expression was confirmed by qRT-PCR (p<0.05). NETs detection revealed a c-di-GMP-dependent trend: PAO1△wspF induced the strongest NETs response (highest immunofluorescence intensity, cf-DNA, and MPO-DNA levels), followed by PAO1 and PAO1/plac-yhjH.

**Conclusions:**

This work reveals how *P.aeruginosa*’s c-di-GMP controls NETs formation in murine pleural infections, bridging bacterial virulence mechanisms with host immune responses. By identifying key regulatory genes, it establishes groundwork for targeted anti-biofilm therapies against chronic infections.

## 1 Introduction

*P.aeruginosa*, a major opportunistic pathogen, exhibits multidrug resistance and environmental adaptability driven by robust biofilm formation (1, 2). Its biofilm-mediated tissue colonization enables chronic infections in immunocompromised hosts, particularly causing refractory pulmonary complications. Globally accounting for 17.6% of culture-positive empyema cases, *P.aeruginosa* shows heightened prevalence in subtropical regions (e.g., Southeast Asia, Middle East), where thermotolerance enhances colonization and nosocomial transmission of resistant strains (3). As a predominant pathogen in pleural infections (empyema/parapneumonic effusions), which feature neutrophilic infiltration, programmed necrosis, and purulent exudate accumulation (4), *P.aeruginosa* exploits pleural microecology to develop recalcitrant biofilms. This biofilm-mediated persistence, coupled with its multidrug resistance profile, escalates therapeutic challenges in treating >80,000 annual US/UK cases, where mortality reaches 10.5% at 30 days and exceeds 19% at one year despite advanced therapies (5).

*P.aeruginosa* employs c-di-GMP signaling to orchestrate biofilm- planktonic transitions, with elevated levels activating exopolysaccharide synthases (e.g., psl/pel) for biofilm fortification and antibiotic resistance, while low concentrations trigger motility genes (flagella/tip pili) enabling dissemination (6–9). This bistable regulation hinges on the Dgc/Pde enzymatic system - diguanylate cyclases (Dgcs) synthesize c-di-GMP whereas phosphodiesterases (Pdes) like YhjH degrade it (10–13). Notably, wspF mutations constitutively activate WspR to hyperaccumulate c-di-GMP, while YhjH-mediated degradation reactivates virulence effectors (e.g., LasB protease) for immune evasion. Through phosphorylation-modulated motor proteins and pilus dynamics, this system enables microenvironmental adaptation, balancing biofilm persistence with invasive dissemination.

Neutrophil extracellular traps (NETs) deploy DNA-histone complexes and antimicrobial proteins (e.g., myeloperoxidase) to establish dual physical/chemical defenses at infection sites. In *P.aeruginosa* models, NETs restrict bacterial dissemination through biofilm structural disruption, effectively blocking invasion into immune-privileged regions including cerebral tissues (14, 15).

Neutrophils are key cellular targets of bacterial c-di-GMP signaling, which regulates their antimicrobial functions across species. While c-di-GMP drives neutrophil recruitment through chemokine pathways (KC/MIP-2/MCP-1) in Klebsiella, Bordetella, and Acinetsobacter infections (13, 16, 17), its mechanistic role in *P.aeruginosa*’s immune evasion remains unexplored, particularly regarding NETsosis regulation.

Understanding how c-di-GMP influences immune evasion mechanisms, particularly through the regulation of NETs formation, is crucial for uncovering the adaptive strategies employed by *P.aeruginosa*. While neutrophils play a vital role in the host defense against bacterial infections, the precise molecular interactions that govern the modulation of NETs by c-di-GMP remain poorly understood.

RNA sequencing (RNA-Seq) transcriptomic analysis is a powerful approach for investigating gene expression changes during bacterial infections (18). In this study, we used RNA-Seq to explore how varying levels of the second messenger c-di-GMP in *P.aeruginosa* influence host gene expression during pleural infection in mice. To identify gene modules with similar expression patterns, we applied the Mfuzz fuzzy clustering algorithm, which categorizes genes based on their expression trends across different conditions (19). Enrichment analysis, along with PPI network analysis, was performed to uncover key regulatory pathways and genes associated with neutrophil extracellular trap (NETs) formation. Furthermore, quantitative real-time PCR (qRT-PCR) was used to validate gene expression levels, while immunofluorescence and ELISA were employed to assess NETs production in each group of mice. This comprehensive approach provides valuable insights into the molecular mechanisms by which c-di-GMP regulates host immune responses during infection. We explore how variations in c-di-GMP levels in *P.aeruginosa* influence the formation of neutrophil extracellular traps (NETs), which play a role in evading immune clearance during pleural infection.

## 2 Materials and methods

### 2.1 Bacterial strains and inoculum preparation

Isogenic *P.aeruginosa* strains-wild-type PAO1, hyper-biofilm mutant PAO1ΔwspF (characterized by elevated intracellular c-di-GMP), and hypo-biofilm mutant PAO1/plac-yhjH (with reduced c-di-GMP levels) were obtained from the Centre for Environmental Life Sciences Engineering, Nanyang Technological University, Singapore (Chua et al., 2013, 2015). All strains were cryopreserved at -80 °C in lysogeny broth (LB; Guangdong Huakai Microbial Technology Co., Ltd., China) supplemented with 25% (v/v) glycerol. For plasmid maintenance, 60 μg/mL tetracycline (Sigma-Aldrich) was added to PAO1/plac-yhjH cultures. Frozen stocks were streaked onto LB agar plates (Beijing Lanqiao Technology Co., Ltd., China) using sterile loops and incubated at 37 °C for 24 h (HPX-400 incubator, Shanghai Yuejin Medical Equipment Co., Ltd.). Single colonies were then inoculated into LB broth and cultured aerobically at 37 °C with 220 rpm agitation for 18 h (THZ-82 shaker, Changzhou Zhibo Instrument Co., Ltd.). Bacterial cells were harvested by centrifugation at 6,000rpm for 3 min (Z366 centrifuge, HERMLE Labortechnik GmbH, Germany), followed by three washes with phosphate-buffered saline (PBS). Bacterial density was standardized to OD600 = 0.1 (10^8 CFU/mL) using a UV-Vis spectrophotometer (T6 model, Beijing Pushi General Instrument Co., Ltd.), with final inocula adjusted to 5×10^7 CFU/mL in sterile PBS for subsequent experiments (20).

### 2.2 Preparation of *P.aeruginosa* reporter strains

*P.aeruginosa* reporter strains PAO1/pcdrA-gfp(PAO1 carrying the pcdrA-gfp report), PAO1ΔwspF/pcdrA-gfp, and PAO1/plac-yhjH/pcdrA-gfp were obtained from the Centre for Environmental Life Sciences Engineering at Nanyang Technological University, Singapore. As reported (21), GFP expression from the pcdrA-gfp reporter positively correlates with intracellular c-di-GMP levels: pcdrA-gfp reporters in *P.aeruginosa*, intracellular c-di-GMP levels positively correlate with GFP expression: high c-di-GMP boosts GFP expression, while low c-di-GMP decreases it. Strains were maintained as described previously, with antibiotic selection as follows (21, 22): 50µg/mL carbenicillin for PAO1/pcdrA-gfp and PAO1ΔwspF/pcdrA-gfp, and 50 µg/mL carbenicillin plus 10 µg/mL tetracycline for PAO1/plac-yhjH/pcdrA-gfp. Bacterial suspensions standardized to OD600 = 0.1 were aliquoted (200 µL/well) into 96-well microplates in six technical replicates and incubated statically at 37°C for 24 h. Biofilm formation was quantified by measuring both optical density (OD600) and GFP fluorescence intensity (ex/em: 485/535 nm) using a Tecan Spark multi-mode microplate reader (Tecan Group Ltd., Austria). Relative fluorescence intensity (RFI) was calculated as RFI/OD600 to normalize reporter activity against bacterial biomass with three biologically independent experiments performed to ensure reproducibility.

### 2.3 Biofilm quantification via crystal violet staining

As previously described, *P.aeruginosa* strains PAO1, PAO1ΔwspF, and PAO1/plac-yhjH were adjusted to 1×10^8 CFU/mL in sterile PBS, and plate coating was performed to verify the colony count. The standardized bacterial suspension (200 µL/well) was aliquoted into 96-well plates (6 technical replicates per strain) and incubated statically at 37°C for 24 h. After incubation, the culture medium in each well was discarded, and wells were washed 3 times with 200 µL sterile PBS, then air-dried at room temperature. Each well was stained with 210 µL of 0.1% crystal violet solution for 30 min, followed by gentle rinsing with deionized water to remove excess stain. After re-drying, 220 µL of absolute ethanol was added to each well, and the plate was incubated with gentle shaking for 30 min to elute the stain. Absorbance was measured at OD595.

### 2.4 Mouse pleural infection model establishment

Nine male C57BL/6J mice (8–10 weeks old, with a body weight of 22–25 g) were obtained from the Laboratory Animal Center of Guangxi Medical University and maintained under specific pathogen-free (SPF) conditions. Mice were randomly allocated into three experimental groups (n=3/group) using a stratified randomization protocol based on body weight: PAO1, PAO1ΔwspF, and PAO1/plac-yhjH. Mice were anesthetized with isoflurane, and aseptic techniques were strictly followed during the subsequent intrathoracic injection procedure: the injection site on the thoracic skin was disinfected with 75% ethanol, and a sterile 30G needle was used to inoculate 100 μL bacterial suspension (5×10^7 CFU, colony-forming unit) at the 7th-8th intercostal space. Using autoclaved surgical instruments, the thoracic cavity was opened gently. Thoracic lavage fluid was collected 24 h post-infection following euthanasia: sterile PBS (0.5 mL) was injected and aspirated twice to maximize fluid recovery, and immediately processed for subsequent analyses. Thoracic lavage fluid samples were serially diluted (10^5^ and 10^6^ in PBS), and 20μL aliquots from each dilution were plated in triplicate on LB agar. Plates were incubated at 37 °C for 18–24 h. *P.aeruginosa* colonization was confirmed by colony morphology. All procedures were approved by the Animal Ethics Committee of Guangxi Medical University (Approval No. 202312014). Mice were allocated into three experimental groups (n=3/group) using a stratified randomization protocol based on body weight: PAO1, PAO1ΔwspF, and PAO1/plac-yhjH. Mice were anesthetized with isoflurane and inoculated via intrathoracic injection of 100μL bacterial suspension (5×10^7 CFU) using a 30G needle at the 7th-8th intercostal space. Thoracic lavage fluid was collected 24 h post-infection following euthanasia: PBS (0.5 mL) was injected and aspirated twice to maximize effusion recovery, and immediately processed for subsequent analyses. Thoracic effusion samples were serially diluted (10^5^and 10^6^ in PBS), and 20μL aliquots from each dilution were plated in triplicate on LB agar. Plates were incubated at 37°C for 18–24 h. The presence of *P.aeruginosa* in the thoracic cavity was confirmed by enumeration of CFU following plating of thoracic lavage fluid. All procedures were approved by the Animal Ethics Committee of Guangxi Medical University (Approval No. 202312014).

### 2.5 Histopathological examination of mouse pleural tissue

At 24 h post-*P.aeruginosa* pleural infection, mice were euthanized by excessive anesthesia, and intercostal parietal pleural tissues were collected. Tissues were fixed in 4% paraformaldehyde (4°C, 24 h), dehydrated in gradient ethanol (70%, 80%, 90%, 100%, 15 min each), cleared in xylene (twice, 10 min each), and paraffin-embedded into 5 μm serial sections. Post-dewaxing to water, sections were stained with hematoxylin (5 min), rinsed to blue with tap water, differentiated in 1% hydrochloric acid-ethanol (30 s), re-rinsed, then eosin-stained (2 min); followed by gradient ethanol dehydration (90%, 100%, 5 min each), xylene clearing (twice, 10 min each), and neutral gum mounting. Pleural pathological changes (inflammatory cell infiltration, tissue damage) were observed via a Nikon Eclipse E100 light microscope, with 5 random fields per group photographed.

### 2.6 Detection of inflammatory markers in mouse pleural lavage fluid via ELISA

For detecting inflammatory cytokines (IL-6, IL-1β, TNF-α) in pleural lavage fluid, specific ELISA kits (JONLNBIO, Mouse IL-6: JL20268; IL-1β: JL18442; TNF-α: JL10484) were used per manufacturers’ protocols. Briefly, pleural lavage fluid was centrifuged at 1000×g for 20 min at 4°C to remove debris, and supernatants were collected. Standards and samples were added to pre-coated microplates, incubated at 37°C, washed 5 times, then HRP-conjugated secondary antibody was added, incubated and re-washed. TMB triggered color development, terminated with termination solution. OD values were measured at 450 nm, and cytokine concentrations were calculated via standard curves.

### 2.7 Biofilm quantification: confocal imaging

To assess biofilm formation *in vivo* and vitro, three *P. aeruginosa* strains were adjusted to an optical density at 600 nm (OD_600_) of 0.1, then statically incubated in a 37 °C incubator for 24 h. Thoracic lavage fluid from *P. aeruginosa*-infected mice (24 h post-infection) was centrifuged at 500 × g for 5 min to remove large debris; 200 μL of the supernatant was incubated on 13 mm×13 mm sterile glass coverslips for 1 h at 37 °C, gently washed with 0.9% NaCl (Solarbio, China) 3 times for 10min, stained with Viable/Dead Kit 30 (Solarbio, China; green: viable, red: dead), imaged using a Leica TCS SP8 STED confocal microscope (oil objective) with Nucgreen (green, Ex 503 nm, Em 530 nm) and EthD-III (red, Ex 530 nm, Em 620 nm) parameters, Z-stack images acquired from base to top, and thickness quantified via LAS X Office 3D plugin (3 fields/sample, 3 replicates, average calculated).

### 2.8 Observation of biofilm in parietal pleural tissue by scanning electron microscopy

At 24 h post-*P.aeruginosa* pleural infection, mice were euthanized by excessive anesthesia; intercostal parietal pleura was excised with autoclaved instruments, immediately fixed in 2.5% glutaraldehyde (4°C, 24 h), washed thrice with 0.1 mol/L phosphate buffer (pH 7.4, 15 min each), secondarily fixed in 1% osmium tetroxide (room temperature, 1–2 h) and re-washed, dehydrated in gradient ethanol (30%-100%, 15–20 min each; 100% twice), replaced with tert-butanol, frozen at -20°C for 30 min, dried via freeze dryer (SCD-350M, Shi’anJia, China), coated with 5–10 nm gold film (ion sputtering coater JY-S100, Guangzhou Jingying, China), and observed under SEM4000X (5 kV) to analyze bacterial aggregation and extracellular matrix deposition.

### 2.9 RNA sequencing and Mfuzz analysis

Mouse pleural lavage fluid was centrifuged (3000rpm, 15 min, 4°C) to separate pellets, which were then washed three times with sterile PBS; RNA was extracted from the pellets using Trizol (Servicebio, China), and its quality was determined (RIN>7.0, OD260/280≥1.8). RNA sequencing of mouse pleural lavage fluid cells (n=3 mice/group) was carried out using Illumina NovaSeq X Plus (150 bp paired-end, Gene Denovo, Guangzhou). Raw reads were quality-controlled first, and clean reads were subjected to bioinformatics analysis via Omicsmart, a dynamic real-time interactive online platform(http://www.omicsmart.com). Gene expression was quantified (FPKM) and differentially expressed genes analyzed (DESeq2, v1.38.3; |log2FC| >2, FDR <0.05). Differentially expressed genes were subjected to clustering analysis using the Mfuzz package, retaining genes with membership values >0.2. These genes were further classified via Mfuzz (fuzzy C-means clustering) to identify co-expression modules.

### 2.10 Protein-protein interaction network construction and functional enrichment analysis

Using Mfuzz package, we identified gene expression trend modules based on dynamic patterns across different c-di-GMP expression levels of *P.aeruginosa.* Persistently downregulated modules were selected, and their genes were used for subsequent PPI networks analysis. These networks were built via STRING (score>0.4). After removing isolated nodes, the network was imported into Cytoscape 3.10.3. The CytoHubba plugin was used to calculate node centrality via Maximum Clique Centrality (MCC), and the top 20 genes by descending scores were selected to build the core subnetwork. Gene clusters exhibiting consistent expression trajectories were functionally annotated using DAVID database for Gene Ontology (GO) terms and Kyoto Encyclopedia of Genes and Genomes (KEGG) database for pathway enrichment. False Discovery Rate (FDR) less than 0.05 was considered statistically significant.

### 2.11 Quantitative real-time PCR validation

Candidate genes from differential expression analysis were validated via quantitative reverse transcription PCR (qRT-PCR). pleural lavage fluid was centrifuged (3000rpm, 15 min, 4°C) to separate pellets, the cell pellet was washed three times with PBS to remove residual supernatant. Briefly, total RNA was extracted from pleural lavage fluid cells using Trizol (Servicebio, China), with quality verified via Nanodrop (Thermo Fisher Scientific, USA; A260/280: 1.8–2.0). cDNA synthesis was performed using a reverse transcription kit (Takara Bio, Inc.).Gene-specific primers were designed and synthesized by Gensys Biotechnology (Shanghai, China). Reactions were conducted in 20μL reactions with 2×SYBR Green Fast qPCR Master Mix (Yifeixue, Nanjing, China), and the thermal cycling protocol followed the manufacturer’s instructions. *Gapdh* was used as the endogenous control for normalization, and relative expression levels were calculated via the 2−ΔΔCt method. Primer sequences are provided in **Supplementary Table S1**.

### 2.12 Primary neutrophil isolation

As previously described, bone marrow neutrophils were isolated from C57BL/6J wild-type mice (23, 24). Briefly, bone marrow cells were isolated from the femurs and tibias via density gradient centrifugation using Histopaque 1119 (Sigma, St. Louis, MO, USA) and Histopaque 1077 (Sigma, St. Louis, MO, USA). The intermediate layer between Histopaque 1119 and Histopaque 1077 was collected, followed by lysis of erythrocytes with RBC lysis buffer (Solarbio, China). All procedures were performed carefully and rapidly on ice under sterile conditions to maintain cell viability and prevent cell activation.

### 2.13 Immunofluorescence protocol for NETs observation

Freshly isolated primary mouse bone marrow neutrophils were resuspended in RPMI 1640(Gibco, USA)medium supplemented with 10% fetal bovine serum(Cellbox, China) and seeded into poly-L-lysine-precoated confocal(Servicebio, China) dishes at 1×10^6^ cells/well. After incubation at 37°C with 5% CO_2_ for 30 min to allow adhesion, three *P.aeruginosa* strains (PAO1 wild-type, PAO1△wspF, PAO1/plac-yhjH) were adjusted to OD600 = 0.1 and added at a multiplicity of infection (MOI) of 100:1; following stimulation at 37 °C with 5% CO_2_ for 3 h, non-adherent bacteria were removed by PBS washing (5 min×3). For immunofluorescence staining (applied to both the above *in vitro*-stimulated neutrophils and *in vivo* mouse pleural effusion samples), cells were fixed with 4% paraformaldehyde (room temperature, 15 min), blocked with 10% goat serum (Solarbio, China; 37 °C, 1 h), and incubated sequentially with primary antibodies (Abcam: anti-Histone H3 (citrulline R2+R8+R17, ab281584, 1:2000); anti-Myeloperoxidase, ab90810, 1:50) at 4°C overnight and fluorescent secondary antibodies (Abcam: goat anti-rabbit IgG H&L (Alexa Fluor^®^ 594, ab150084); goat anti-mouse IgG H&L (Alexa Fluor^®^ 488, ab150117); both 1:400) at 37°C for 1 h in the dark. After PBS washing (5 min×3), cells were stained with DAPI (Solarbio, China) for 10 min in the dark, and images were acquired using a Leica TCS SP8 STED confocal microscope (excitation wavelengths: 405 nm for DAPI, 488 nm for Alexa Fluor^®^ 488, 594 nm for Alexa Fluor^®^ 594). Under a laser confocal microscope with an oil immersion objective, 5 non-overlapping fields were randomly selected per group. Total neutrophils were defined as MPO^+^DAPI^+^ cells. NETs-positive cells were identified as cells with nuclear fragmentation and extracellular fibrillar citH3^+^MPO^+^ colocalization (excluding intracellular-only signals). The percentage of NET-releasing cells was calculated as (number of NETs-positive cells/total neutrophils) × 100%.

### 2.14 Measurement of MPO-DNA complexes and cf-DNA in pleural effusion

The concentration of myeloperoxidase-DNA (MPO-DNA) complexes in murine pleural effusion supernatants was quantified using a Mouse MPO-DNA Complex commercial enzyme-linked immunosorbent assay (ELISA) kit (F31066-A, FANKEW, Shanghai, China), Cell-free DNA (cfDNA) was quantified using the Quant-iT™ PicoGreen™ dsDNA Assay Kit (Cat. No. P7589, Invitrogen, USA) according to the manufacturer’s instructions.

### 2.15 Statistical analysis

All quantitative data are presented as mean ± standard deviation (SD). Intergroup differences were analyzed using one-way analysis of variance (ANOVA) followed by Tukey’s *post hoc* test for normally distributed data with homogeneous variance; for non-normal data, the Kruskal-Wallis H test was used, with Dunn’s test (Bonferroni-corrected) for pairwise comparisons when significant overall differences were found. All analyses were implemented in GraphPad Prism version 9.5. Statistical significance was defined as p <0.05.

## 3 Results

### 3.1 Comparison of biofilms and c-di-GMP levels *in vitro*

*In vitro* analyses of three *P. aeruginosa* strains (**Supplementary Figures S1A, B**) showed: via pcdrA-gfp reporter, PAO1△wspF had the strongest GFP fluorescence and highest c-di-GMP, followed by wild-type PAO1 and PAO1/plac-yhjH; via crystal violet staining (**Supplementary Figure S1C**), their biofilm biomass exhibited the same hierarchy (PAO1△wspF > PAO1 > PAO1/plac-yhjH), with significant pairwise differences for both parameters. These trends matched confocal microscopy results of their *in vitro* biofilms (**Supplementary Figure S2**): PAO1△wspF formed the densest biofilms, followed by wild-type PAO1, and PAO1/plac-yhjH the least.

### 3.2 Pleural histopathology

**Figure 1:**
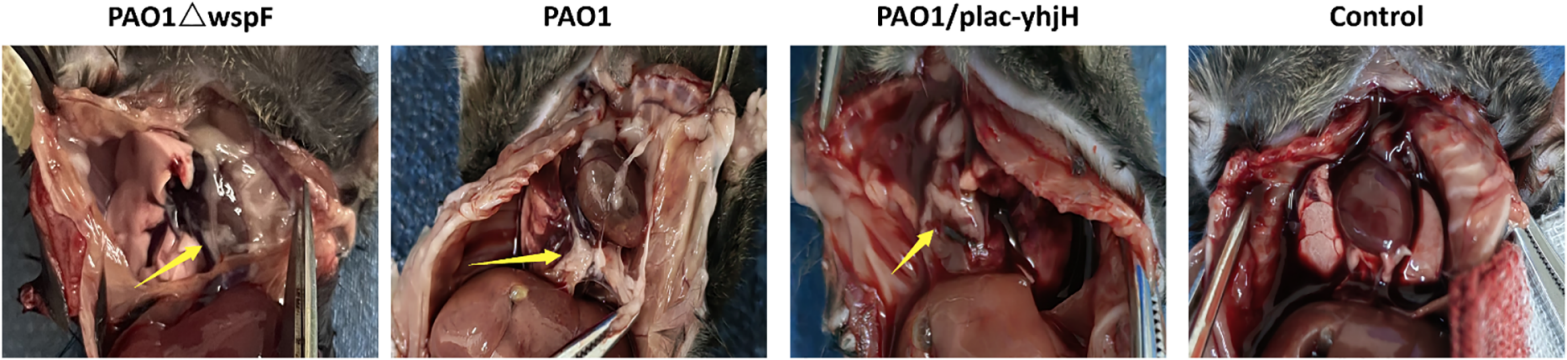
Anatomical observations of mice infected with different *P.aeruginosa* strains. Mice were randomly assigned to four groups (n=3 per group) and inoculated with *P. aeruginosa* PAO1ΔwspF, PAO1, PAO1/plac-yhjH, or sterile PBS (Control). Anatomical dissection was performed at 24 h post-inoculation. Purulent exudates are marked by yellow arrows.

**Figure 2:**
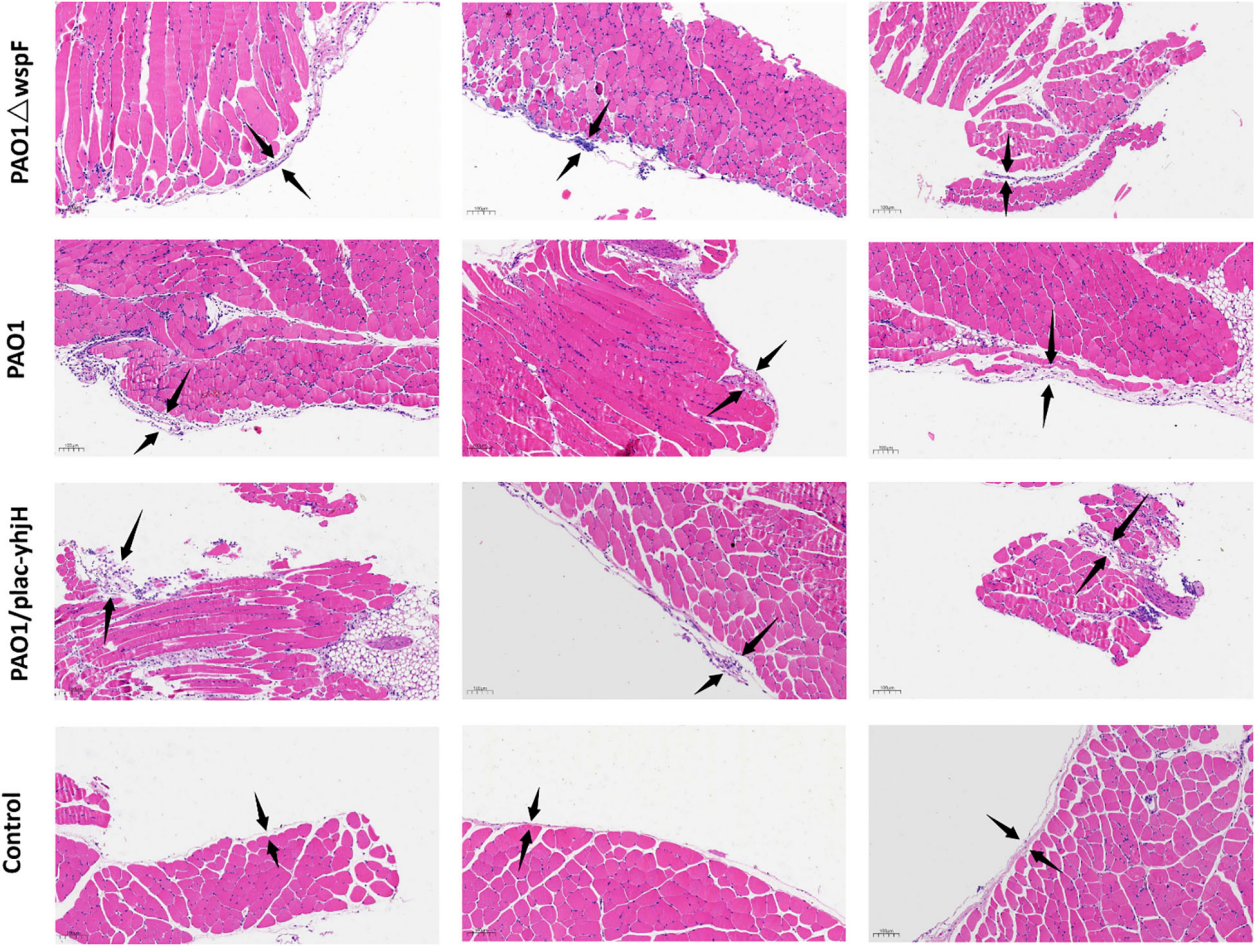
Morphological changes of the parietal pleura (H&E staining, 100×), scale bars represent 100μm. Comparison between PBS control group and *P.aeruginosa*-infected groups (PAO1, PAO1△wspF, PAO1/plac-yhjH, n=3 per group) and morphological differences among infected groups. The region between the two black arrows denotes the pleura.

**Figure 1** shows representative thoracic cavity dissection images of mice in different groups: the *P. aeruginosa*-infected groups (including PAO1, PAO1△wspF, and PAO1/plac-yhjH strains) had obvious purulent secretions, while the PBS control group had none. In HE-stained sections (**Figure 2**), compared with the PBS control group, the *P. aeruginosa-*infected groups showed obvious parietal pleura thickening, accompanied by extensive inflammatory cell infiltrations. The control group exhibited a clear and thin pleural structure with regularly arranged cells and no obvious inflammatory cell aggregation; in contrast, the infected groups presented increased pleural tissue thickness, with massive inflammatory cell infiltrations in the interstitium, suggesting that *P. aeruginosa* infection could induce pleural inflammatory responses and histomorphological changes. However, no morphologic differences identifiable by naked eye were observed among the three *P. aeruginosa*-infected groups.

### 3.3 Observation of biofilm formation after *P.aeruginosa* pleural infection in mice (verification by CLSM and SEM)

**Figure 3:**
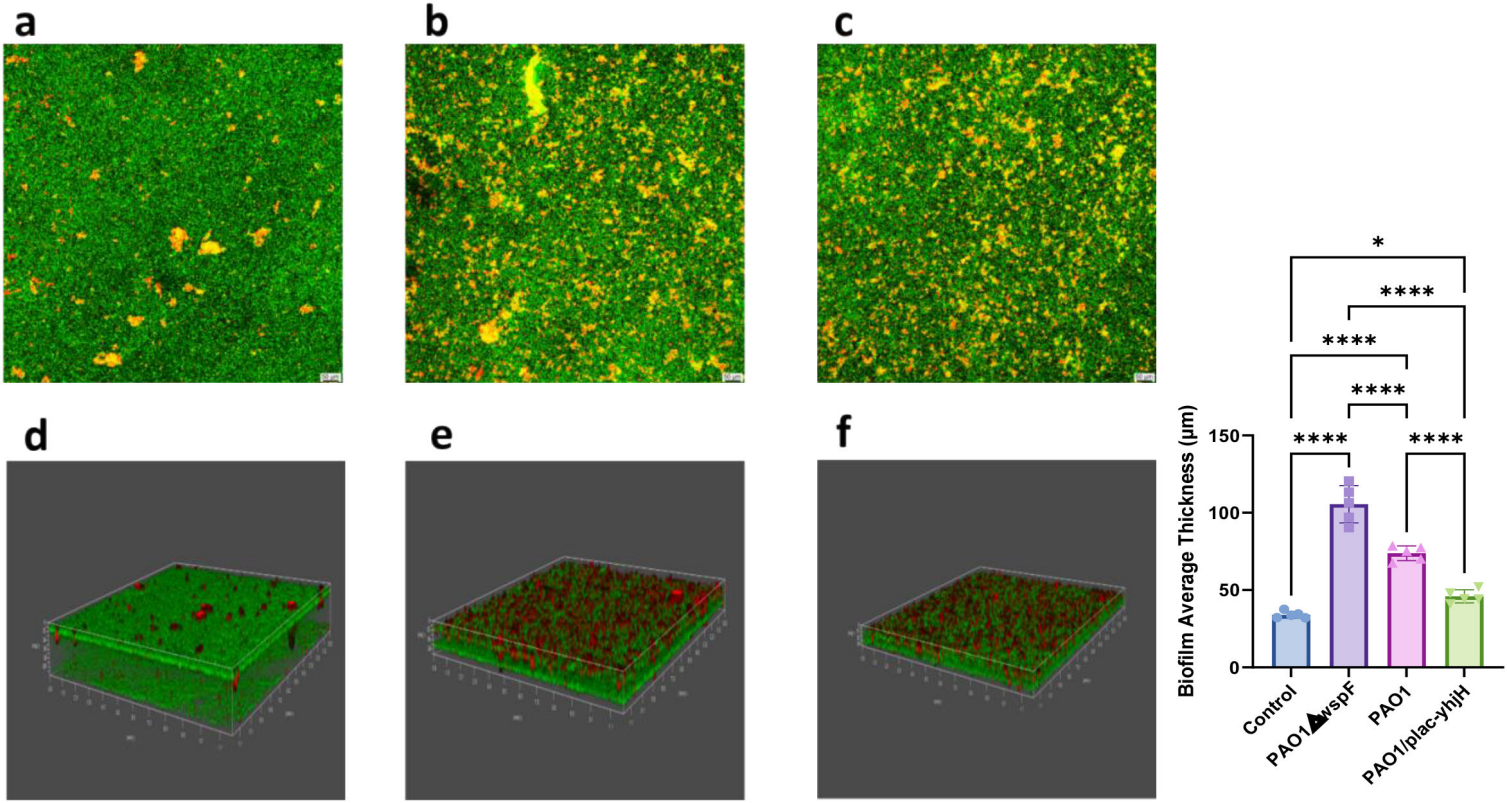
Comparison of biofilms from three *P. aeruginosa* strains in pleural lavage fluid via confocal microscopy. Fluorescence labels: green: viable bacteria; red: dead bacteria. **(A)** PAO1△wspF biofilm fluorescence 2D imaging; **(B)** PAO1 biofilm fluorescence 2D imaging; **(C)** PAO1/plac-yhjH biofilm fluorescence 2D imaging; **(D)** PAO1△wspF biofilm 3D reconstruction; **(E)** PAO1 biofilm 3D reconstruction; **(F)** PAO1/plac-yhjH biofilm 3D reconstruction. Five non-overlapping fields were randomly selected per group (n=3). The data are presented as mean ± SD, scale bars represent 50μm. *p < 0.05, ****p < 0.0001.

**Figure 4:**
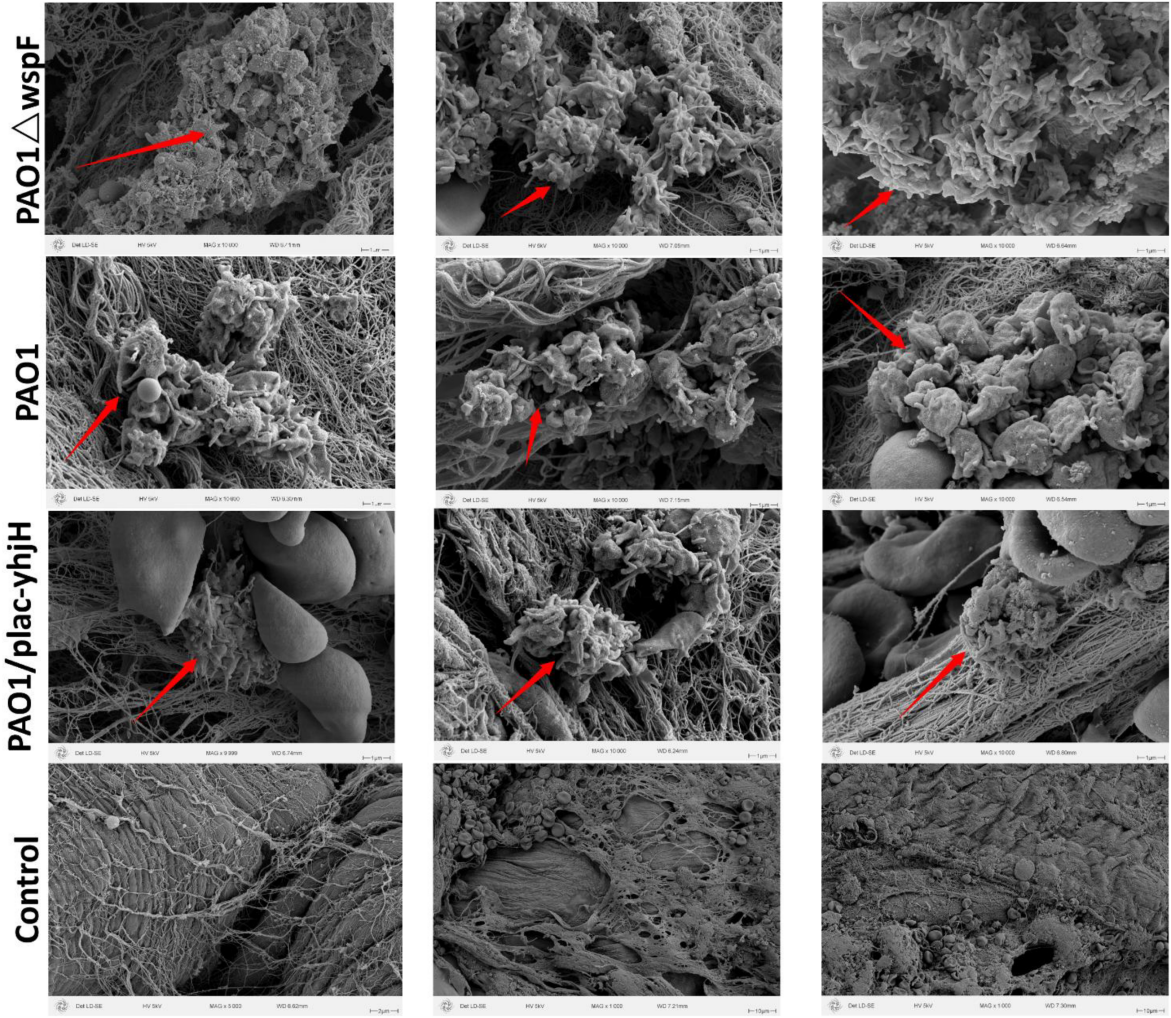
SEM of bacterial aggregation and biofilm formation in murine pleural tissues. Images show the pleural surface at 10,000× magnification. *P.aeruginosa* was embedded in an electron-dense extracellular matrix, forming obvious biofilm-like structures. Biofilms have been labeled with red arrows.

Quantitative analysis via CLSM (**Figure 3**) revealed obvious hierarchical differences in biofilm thickness among the tested strains: the PAO1ΔwspF strain formed the thickest biofilms, followed by the wild-type PAO1 strain, while the PAO1/plac-yhjH strain formed the thinnest biofilms. This result was further confirmed by SEM observation of pleural tissues (**Figure 4**): the degree of bacterial aggregation and the pattern of extracellular matrix deposition in SEM images were completely consistent with the order of biofilm formation quantified by CLSM (PAO1ΔwspF > PAO1 > PAO1/plac-yhjH). Specifically, the PAO1ΔwspF strain formed dense bacterial aggregates on the surface of pleural tissues, surrounded by a large amount of flocculent extracellular matrix; in contrast, only scattered bacteria were observed in the PAO1/plac-yhjH strain group, with extremely low content of extracellular matrix. Collectively, the morphological evidence from CLSM and SEM confirmed that the infection process of *P.aeruginosa* in the mouse pleural cavity completed the transition from a planktonic state to a biofilm-associated infection state within 24 hours.

### 3.4 Pro-Inflammatory cytokines’s expression in murine pleural lavage fluid

At 24 h post *P. aeruginosa* infection, ELISA quantified IL-6, IL-1β, and TNF-α in pleural effusion (**Supplementary Figure S3**). Compared to the Control group, all infected groups (PAO1△wspF, PAO1, PAO1/plac-yhjH) showed significantly elevated IL-6, with a gradient of PAO1/plac-yhjH > PAO1 > PAO1△wspF. IL-1β was negligible in Controls but markedly increased in all infected groups; PAO1 and PAO1/plac-yhjH had similar IL-1β levels but both were significantly higher than PAO1△wspF (P < 0.0001). For TNF-α, Controls showed no significant differences from PAO1△wspF or PAO1/plac-yhjH, while PAO1 had significantly higher TNF-α than both, with no difference between PAO1△wspF and PAO1/plac-yhjH.

### 3.5 Differential gene expression in mice infected with *P.aeruginosa* at different c-di-GMP levels

**Figure 5:**
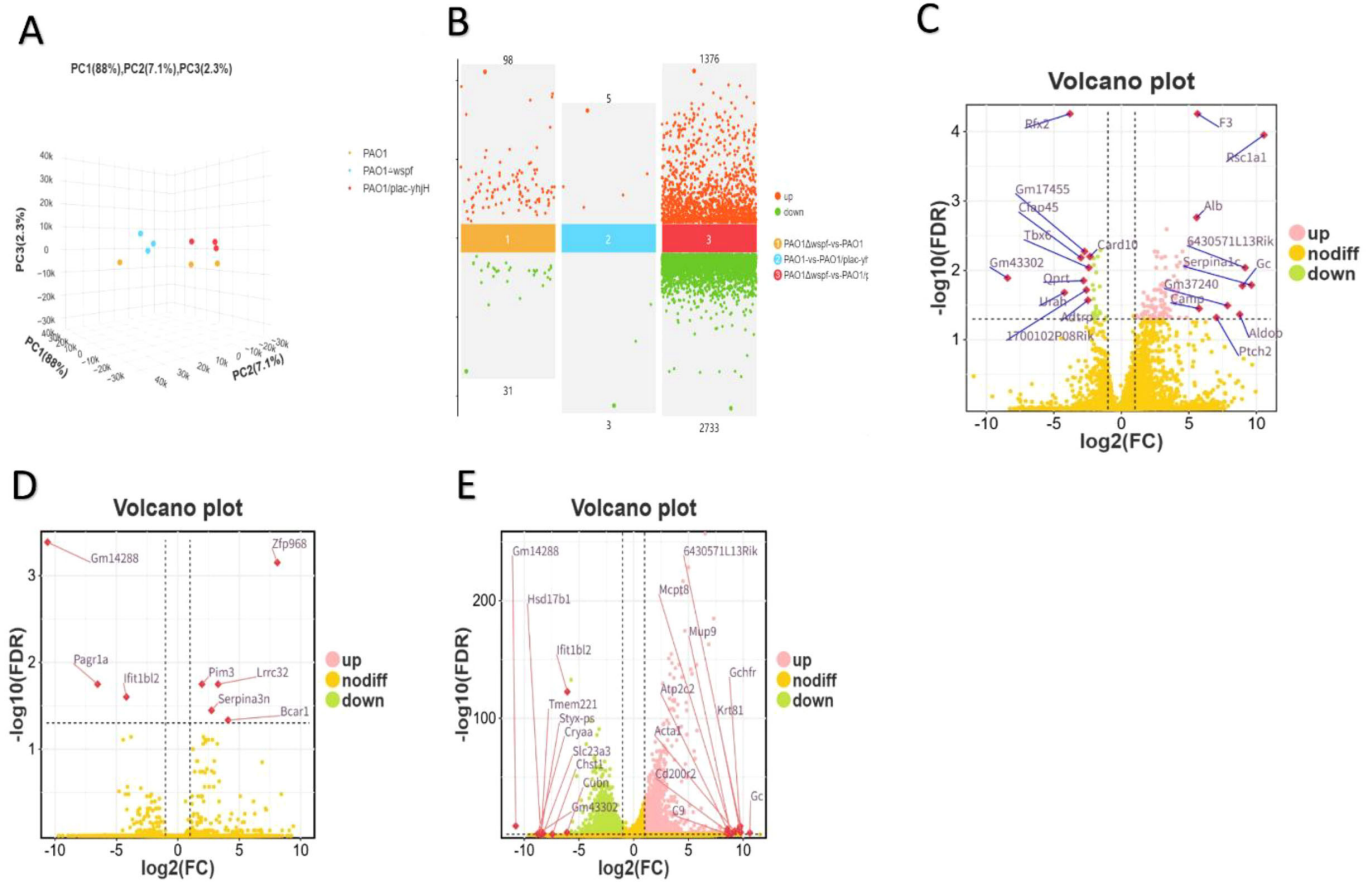
Differential gene expression in mice infected with *P.aeruginosa* at different c-di-GMP levels. **(A)** The PCA plot illustrates the distribution of mRNA expression profiles among different infection groups, including PAO1, PAO1△wspF, and PAO1/plac-yhjH. The analysis captures variance across the first three principal components (PC1: 88%, PC2: 7.1%, and PC3: 2.3%). **(B)** Multi-group differential scatter plots reveal significant differential gene expression. Specifically, 98 upregulated and 31 downregulated genes were identified in PAO1△wspF vs. PAO1, 5 upregulated and 3 downregulated in PAO1 vs. PAO1/plac-yhjH, and 1376 upregulated and 2733 downregulated in PAO1△wspF vs. PAO1/plac-yhjH (FDR < 0.05, |log_2_FC| > 2). **(C–E)** Volcano plots of differential gene expression between WT-wspF vs. WT-PAO1 **(C)**, WT-PAO1 vs. WT-yhjH **(D)**, and WT-wspF vs. WT-yhjH **(E)**, respectively (FDR < 0.05, |log_2_FC| > 2). Genes are sorted by log_2_FC values, and the top 10 upregulated and downregulated genes are labeled in the plots.

**Figure 6:**
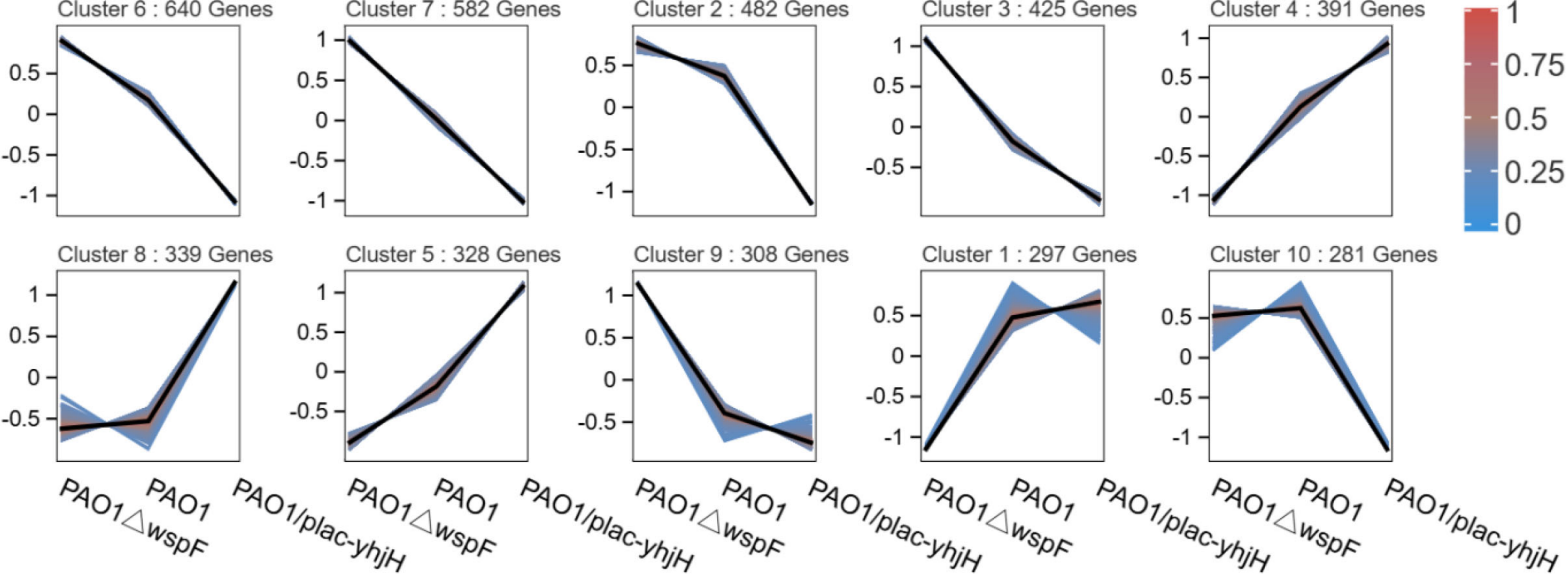
mRNA clustering of infected mice based on Mfuzz analysis. The black lines represent the median expression trend of each cluster, while the colored areas indicate the membership degree of genes within the cluster from 0 (blue) to 1 (red), reflecting how strongly a gene belongs to the cluster). The genes were grouped into three major expression trends: downregulated clusters (Cluster (2,3,6,7,9), upregulated clusters (Cluster 1, 5, 4, 8), fluctuating clusters (Cluster 10).

Through integrated RNA sequencing and bioinformatics analyses, we systematically characterized host transcriptomic responses using principal component analysis (PCA), volcano plots, and functional enrichment strategies. Comparative transcriptome profiling identified c-di-GMP-dependent regulatory networks modulating host-pathogen interactions, with particular emphasis on biofilm-associated immune evasion mechanisms and metabolic reprogramming events. PCA was performed to assess the overall gene expression profiles of *P.aeruginosa* strains in mice with varying levels of c-di-GMP. The three experimental groups, namely PAO1, PAO1ΔwspF, and PAO1/plac-yhjH, showed distinct clustering in the 3D PCA plot (**Figure 5A**). The first principal component (PC1) accounted for 88% of the variance, indicating a strong separation between the strains. PAO1 (yellow) clustered separately from PAO1ΔwspF (blue) and PAO1/plac-yhjH (red), suggesting significant differences in gene expression across these strains. Differential gene expression analysis (**Figure 6B**) revealed a significant number of differentially expressed genes (DEGs) between the experimental groups. In the comparison between PAO1ΔwspF and PAO1 (Panel 1), 98 genes were upregulated (orange dots) and 31 genes were downregulated (green dots). In the comparison between PAO1 and PAO1/plac-yhjH (Panel 2), 5 genes were significantly upregulated, while 3 genes were downregulated. Finally, the comparison between PAO1ΔwspF and PAO1/plac-yhjH (Panel 3) identified a substantial number of DEGs, with 1376 genes upregulated and 2733 genes downregulated (FDR < 0.05, |log2FC| > 2). Volcano plots display differential gene expression between WT-wspF vs WT-PAO1 (**Figure 5C**), WT-PAO1 vs WT-yhjH (6D), and WT-wspF vs WT-yhjH (6E) (FDR < 0.05, |log_2_FC| > 2), with the top 10 upregulated and downregulated genes labeled. These results indicate that varying levels of c-di-GMP in P. aeruginosa significantly influence the host gene expression profile in mouse thoracic lavage fluid, highlighting the regulatory role of *P. aeruginosa*’s c-di-GMP in shaping host gene expression under pleural infection conditions.

### 3.6 mRNA clustering of infected mice based on Mfuzz analysis

Differential gene expression analysis revealed significant transcriptional remodeling across PAO1ΔwspF, PAO1, and PAO1/plac-yhjH groups. To further investigate the interrelationships among the three groups, we conducted Mfuzz clustering analysis to delineate dynamic gene expression patterns. Mfuzz clustering identified 10 distinct gene clusters across the three infection groups (PAO1△wspF, PAO1, and PAO1/plac-yhjH) (**Figure 6**). Differential gene expression analysis revealed significant transcriptional remodeling across the PAO1ΔwspF, PAO1, and PAO1/plac-yhjH groups. To further investigate the interrelationships among these three groups, we conducted Mfuzz clustering analysis (membership>0.2) to delineate dynamic gene expression patterns. This analysis identified 10 distinct gene clusters, among which 5 clusters (Clusters 2, 3, 6, 7, 9) exhibited consistent and sustained downregulated expression trends-results that highlight dynamic changes in mRNA expression profiles driven by differential c-di-GMP levels.

### 3.7 KEGG and GO enrichment analyses

**Figure 7:**
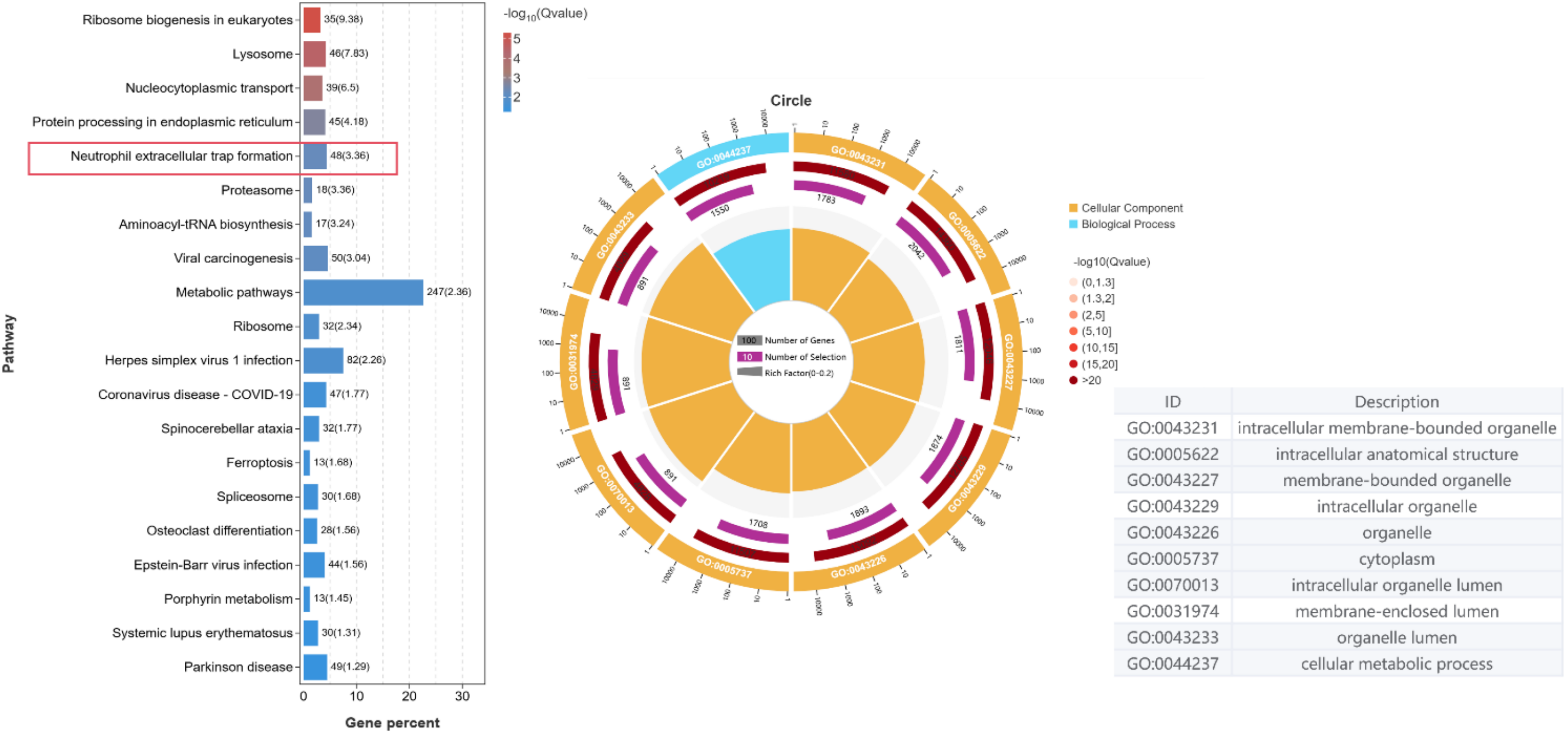
KEGG and GO enrichment analyses. **(A)** KEGG pathway analysis: Top 20 pathways from the Kyoto Encyclopedia of Genes and Genomes, based on genes from the five coordinately downregulated modules (Clusters 2, 3, 6, 7, 9) that are associated with *P. aeruginosa* pleural infection. **(B)** GO enrichment circular plot: Circular plot visualizing the top 10 enriched biological processes from Gene Ontology (GO) analysis of genes in the five coordinately downregulated modules (Clusters 2, 3, 6, 7, 9 from Mfuzz Analysis). Each segment corresponds to a biological process, with arc length proportional to the percentage of involved genes. Color gradient (dark blue to light yellow) indicates significance (FDR < 0.05); radial labels show process names and gene percentages.

To understand how host cells isolated from thoracic lavage fluid respond to c-di-GMP modulation, we went on to carry out functional enrichment analysis on five modules that were coordinately downregulated - Clusters 2, 3, 6, 7, and 9 from the Mfuzz Analysis. These modules showed a sustained downward trend, which aligned with the decreasing c-di-GMP levels. **Figure 7** depicts the KEGG pathway and Gene Ontology (GO) enrichment analyses of genes associated with neutrophil extracellular traps (NETs) formation, exploring the impact of *P.aeruginosa* c-di-GMP concentration on mouse NETs stimulation. In the KEGG Pathway Enrichment results (**Figure 7A**), the ‘Neutrophil extracellular trap formation’ pathway was significantly enriched, with 48 contributing gene. This finding highlights its importance in the host’s immune response to *P. aeruginosa* infection. Additionally, ‘Ribosome biogenesis in ‘eukaryotes’ and ‘Lysosome’ pathways were enriched, indicating the complexity of the underlying regulatory network. A circular plot was used to illustrate the gene count and percentage of different pathways. The GO enrichment results (**Figure 7B**) further support the structural and metabolic mechanisms of NETs: enrichment of terms like ‘intracellular membrane-bounded organelle’ indicates the involvement of organelles such as lysosomes (key for storing and releasing granular enzymes essential for chromatin decondensation and NETs structural integrity) and the endoplasmic reticulum (involved in post-translational modification of NETs-related proteins) in NETs formation. Enrichment of ‘metabolic pathways’ and ‘cellular metabolic process’ suggests metabolic reprogramming fuels NETs biogenesis, as neutrophils undergo rapid metabolic shifts to meet the energy and biosynthetic demands of chromatin remodeling, granular protein synthesis, and membrane trafficking during NETs extrusion. The NETs formation pathway plays a pivotal role in regulating the immune response and the dynamic interaction between bacterial c-di-GMP expression and host defense mechanisms in the mouse infection model. The KEGG and GO enrichment analyses have uncovered important functional genes, biological processes, and cellular components associated with NETs formation, emphasizing its significance in immune regulation during *P.aeruginosa* infection.

### 3.8 Hub genes in NETs formation identified by PPI network

**Figure 8:**
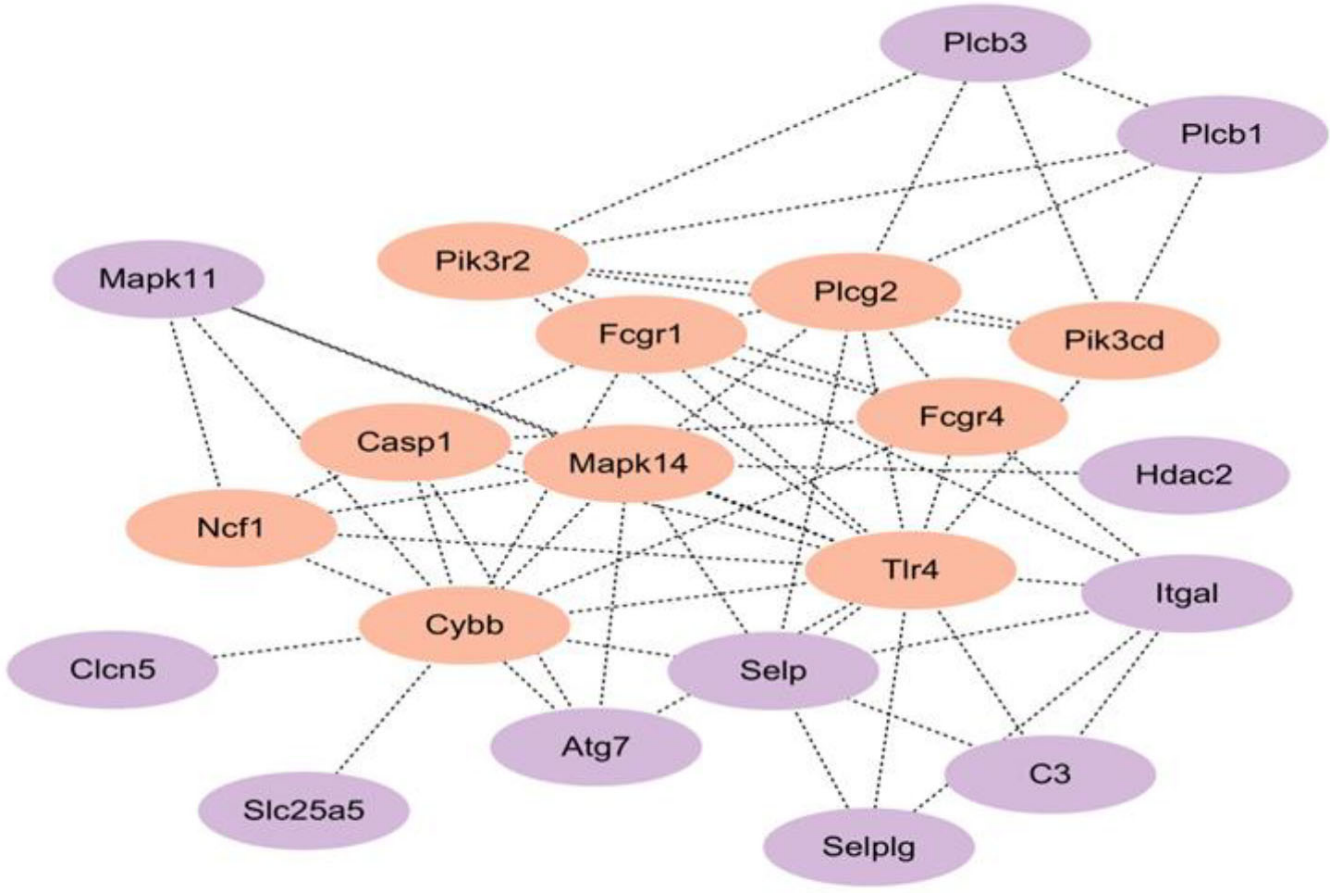
Hub genes in NETs formation identified by PPI network. The PPI network, constructed from genes in the five coordinately downregulated modules (Clusters 2, 3, 6, 7, 9 from Mfuzz analysis), identified 20 hub genes involved in the analyzed biological processes. Among them, the top 10 ranked hub genes, highlighted in orange, include *Mapk14*, *Pik3cd*, *Cybb*, and others; the remaining 10 were highlighted in purple.

Among these five coordinately downregulated modules from Mfuzz analysis (Clusters 2, 3, 6, 7, 9), the top 10 hub genes (*Pik3cd, Ncf1, Pik3r2, Fcgr4, Fcgr1, Plcg2, Casp1, Mapk14, Cybb, and Tlr4)* are highlighted in orange in **Figure 8**. These genes are mainly involved in key pathways that regulate inflammatory responses and immune activation, including NF-κB, MAPK, and PI3K-Akt signaling. Representative genes such as Mapk14, Tlr4, and Cybb, which have high connectivity, play central roles in immune response and signal transduction. The remaining 10 key genes (Mapk11, Clcn5, Slc25a5, Atg7, Selp, Selplg, C3, Hdac2, Plcb1, Plcb3) are highlighted in purple, with involvement in complementary pathways including p38 MAPK signaling, autophagy, complement activation, calcium signaling, and epigenetic regulation, which collectively support processes like immune cell recruitment, metabolic homeostasis, and transcriptional control of NETs formation. Collectively, these results shed light on the critical molecular interactions driving NETs formation and identify potential targets for follow-up investigations.

### 3.9 qRT-PCR validation of *Mapk14*, *Cybb*, *Pik3cd* expression

**Figure 9:**
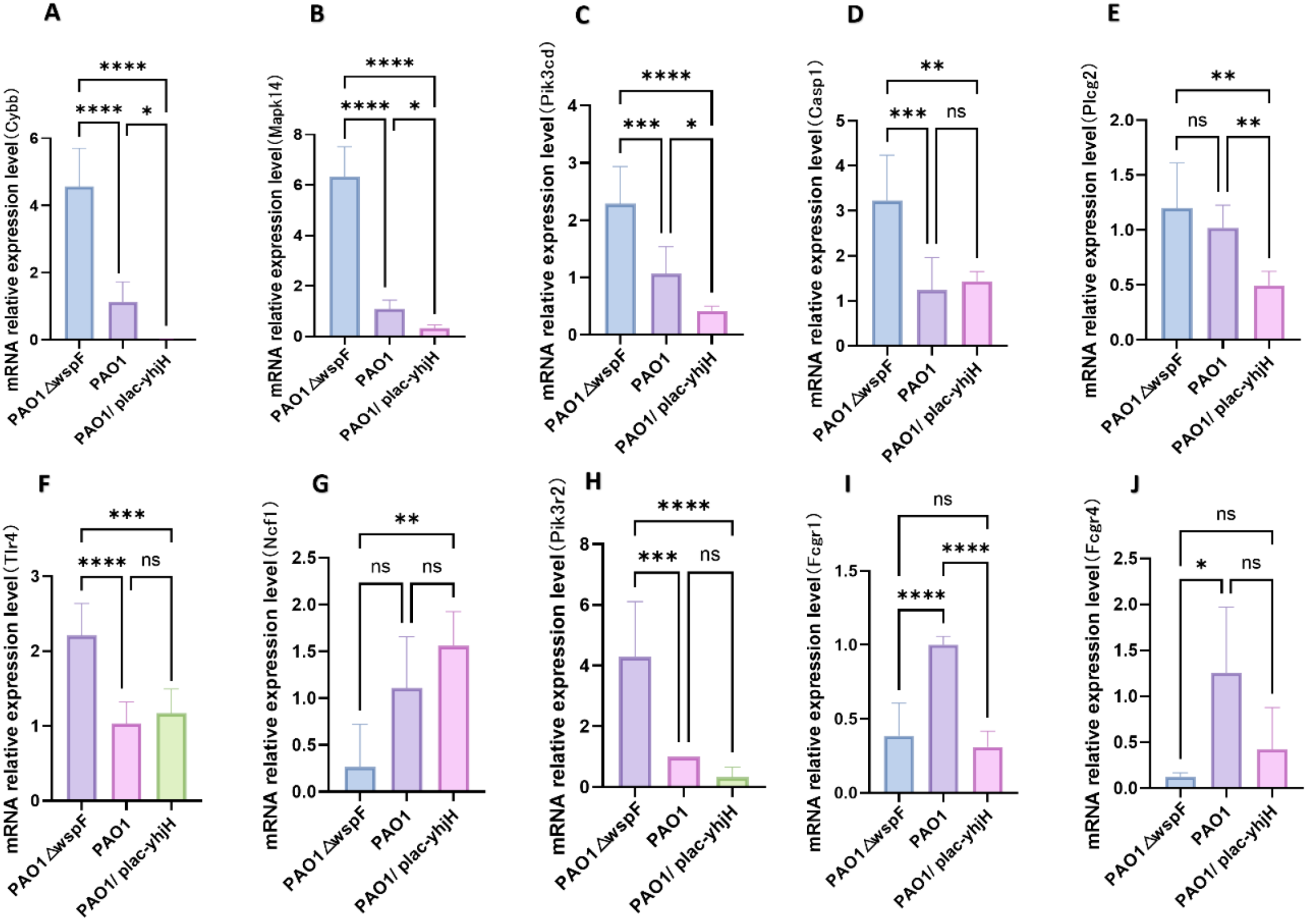
Validation of hub genes identified by PPI network analysis via qRT-PCR. **(A–J)** Demonstrate the expression of *Cybb*, *Mapk14*, *Pik3cd*, *Casp1*, *Plcg2*, *Tlr4*, *Ncf1*, *Pik3r2*, *Fcgr1*, *Fcgr4*.Data are expressed as means ± standard deviation. *p < 0.05, **p < 0.01, ***p < 0.001, ****p < 0.0001, ns, no significance.

Gene expression levels were analyzed using one-way ANOVA to identify significant differences among experimental groups. qRT-PCR validation of prioritized candidate genes (orange-highlighted) revealed *Cybb*, *Mapk14*, and *Pik3cd* as significantly differentially expressed (p < 0.05) across pairwise comparisons of *P.aeruginosa* infection groups (PAO1, PAO1△wspF, PAO1/plac-yhjH) (**Figure 9**). One-way ANOVA confirmed group-wide transcriptional dynamics, with expression patterns tightly correlated to bacterial c-di-GMP levels, implicating their roles in c-di-GMP-dependent neutrophil activation and immune regulation during host-pathogen interplay.

### 3.10 Comparison of NETs formation between the PAO1△wspF, PAO1, and PAO1/plac-yhjH

**Figure 10:**
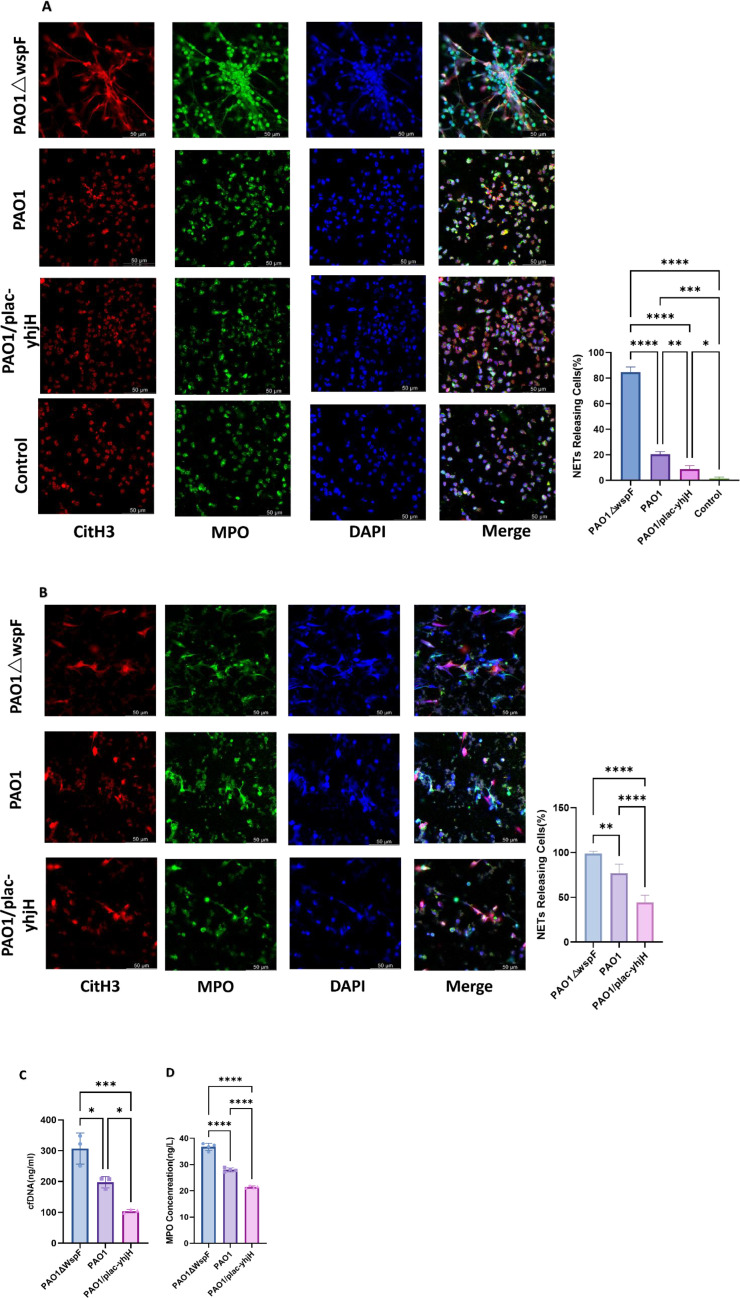
Comparison of NETs formation between the PAO1△wspF, PAO1,and PAO1/plac-yhjH. **(A)***In vitro*: Primary mouse bone marrow neutrophils (n=3 per group) co-incubated with *P.aeruginosa* (PAO1ΔwspF, PAO1, PAO1/plac-yhjH, MOI = 100:1), stained with DAPI (blue), citrullinated H3 (red), MPO (green); percentage of NETs-releasing cells were measured. **(B)***In vivo*: Pleural lavage fluids from mice infected with the three strains, stained with DAPI (blue), citrullinated H3 (red), MPO (green); percentage of NETs-releasing cells were measured. Uninfected control not shown (scarce immune cells in lavage fluid). **(C)** cf-DNA levels in the pleural effusion of each group. **(D)** MPO levels in the pleural effusion of each group. Data are expressed as means ± standard deviation. *p < 0.05, ** *p* < 0.01, *** *p* < 0.001, **** *p* < 0.0001.

Immunofluorescence analysis, both *in vitro* (primary bone marrow neutrophils) and *in vivo* (pleural effusion samples) (**Figure 10A**), revealed distinct patterns of NETs formation among different *P.aeruginosa* strains. NETs were visualized by co-staining extracellular DNA (DAPI, blue), citrullinated histone H3 (red), and myeloperoxidase (MPO, green).Notably, the order of NETs formation intensity was consistent in both *in vitro* and *in vivo* models: PAO1△wspF > PAO1 > PAO1/plac-yhjH. Quantitative analysis (**Figure 10B**) further confirmed statistically significant differences among the groups (p < 0.05), with the PAO1△wspF strain known to form more biofilm, inducing the strongest NETs response. Additionally, cell-free DNA (cf-DNA) levels (**Figure 10C**) and MPO concentrations (**Figure 10D**) in the supernatants of centrifuged pleural lavage fluids from infected mice showed a parallel trend: the PAO1△wspF group exhibited the highest levels, followed by the PAO1 group, while the PAO1/plac-yhjH group had the lowest. These findings collectively indicate a direct correlation between bacterial c-di-GMP levels and NETs induction, suggesting that c-di-GMP in *P.aeruginosa* modulates the activation of host neutrophil-mediated immune responses. It should be noted that due to the scarcity of immune cells in pleural lavage fluids from non-infected mice, a PBS control group was not included in the *in vivo* immunofluorescence analysis.

## 4 Discussion

This study investigates how *P.aeruginosa* regulates biofilm formation through cyclic di-guanosine monophosphate (c-di-GMP) and its subsequent impact on the formation of neutrophil extracellular traps (NETs) in the host. By constructing *P.aeruginosa* strains with different c-di-GMP expression levels (PAO1△wspF, PAO1, and PAO1/plac-yhjH) and analyzing their transcriptomes in a mouse thoracic infection model, we clarified the key role of c-di-GMP in bacterial pathogenicity and host immune response.

Transcriptomic analysis revealed different gene expression patterns among the three strains. Differential expression and clustering analysis identified five gene clusters that were consistently downregulated in the infection group, which may be related to bacterial adaptation to host immune challenges. Kyoto Encyclopedia of Genes and Genomes (KEGG) enrichment analysis highlighted 48 genes significantly associated with NETs-related signaling pathways, indicating that c-di-GMP can regulate NETs formation during infection.

As a key host defense mechanism, NETs can restrict the spread of pathogens and enhance local immune responses. Our research results indicate that c-di-GMP exerts a dual regulatory role by altering the expression of NETs-related genes. PPI network analysis identified key hub genes (*Mapk14*, *Cybb*, and *Pik3cd*) involved in NETs formation, which may affect inflammatory signaling and immune cell activation.

From the perspective of the mechanism by which bacterial virulence factors and biofilms synergistically regulate NETs, the biofilm production of the high c-di-GMP strain PAO1△wspF is significantly increased, and changes in the expression of bacterial virulence factors during biofilm formation may be the key to the differences in NETs-inducing ability. Previous studies have confirmed in a *P.aeruginosa* corneal infection model that the type III secretion system (T3SS) is highly expressed at the bottom of the biofilm, and its effector proteins (such as ExoS) can directly activate the NETs pathway of neutrophils, restricting bacterial diffusion while promoting biofilm stability (25). In this study, due to the increased level of c-di-GMP, the PAO1△wspF strain not only has a more complex biofilm structure but may also regulate the expression of T3SS-related genes (such as pcrV) through c-di-GMP, further enhancing the activation effect on neutrophils, ultimately leading to a significant increase in NETs production. This is consistent with the mechanism of ‘biofilm-associated virulence factors driving NETs production’ (25). In addition, the interaction between the extracellular matrix of *P.aeruginosa* biofilms and NETs has been confirmed to be an important link in regulating the effects of NETs. Review studies have pointed out that extracellular polysaccharides of biofilms (such as Psl and Pel) can bind to the DNA skeleton or antimicrobial proteins of NETs through charge interactions, affecting the bactericidal efficiency of NETs and the stability of biofilms (26). In this study, through KEGG pathway enrichment analysis, it was found that 48 differential genes were significantly enriched in the neutrophil extracellular trap formation pathway, suggesting that c-di-GMP may indirectly affect the activation of NETs production-related pathways by regulating the expression of biofilm-related genes (such as polysaccharide synthesis genes). This finding further supplements the theoretical framework of the biofilm-NETs interaction-dependent gene regulatory network.

From the perspective of the association between key regulatory genes and signaling pathways, among the 10 hub genes identified through the PPI network in this study, the differential expressions of *Cybb, Mapk14, and Pik3cd* (qRT-PCR, p<0.05) provide a molecular-level explanation for the regulation of NETs by c-di-GMP. *Mapk14* encodes p38α mitogen-activated protein kinase, which is a central node in inflammatory signal transduction. Its phosphorylation activity directly regulates the nuclear translocation of transcription factors related to NETs (such as NF-κB) (27). High *Mapk14* expression in PAO1△wspF-infected mice may stem from high c-di-GMP-mediated strengthening of PAO1△wspF’s host-stimulating signals, which activates pattern recognition receptors and upregulates this key p38 Mapk to reinforce defenses like phagocytosis (28). *Cybb* encodes *NOX2*, a key molecule for NETs formation. The reactive oxygen species (ROS) produced by NOX2 can trigger chromatin release and enhance the antibacterial efficacy of NETs (29). Deficiency of Cybb impairs NETs generation (30),which is consistent with previous studies: *P.aeruginosa* biofilms in diabetic wound infections exacerbate ROS accumulation, forming an oxidative stress cycle (31). Additionally, Cybb is a core NETs-related gene in sepsis, and its high expression predicts poor prognosis (32). P*.aeruginosa* with high c-di-GMP can prompt the host to upregulate *Cybb* in response to ROS stress by enhancing biofilms (c-di-GMP regulates biofilm development (33)), which is consistent with the conclusion of Dömer et al. (30) that *Cybb* expression is related to oxidative stress in severe infections; while fibronectin intervention can reduce sepsis damage and decrease *Cybb* transcription levels (34), and Qi et al. (32) also found that blocking the glyoxylate cycle of *P.aeruginosa* under chlorine stress conditions can cause the bacteria to enter a Viable But Non-Culturable (VBNC) state due to ROS accumulation. In summary, *Cybb* is both a driver of NETs formation and a potential therapeutic target for severe infections. Finally, the key role of *Pik3cd* (PI3Kδ) in immune cell regulation has been confirmed. As a catalytic subunit of phosphatidylinositol 3-kinase (PI3K), *Pik3cd* regulates the survival and activation of neutrophils through the AKT/mTOR pathway (35, 36). Aji et al. (37) reported that activation of the PI3K/MAPK/AKT axis drives NETs-mediated pathological processes in *P.aeruginosa*-induced lung injury. Our study extends this finding by identifying PI3KCD (the δ subtype of the PI3K catalytic subunit) as a key regulator of c-di-GMP-modulated NETs formation during thoracic infections, emphasizing the subtype-specific role of PI3K in bacterial-host interactions.

From the biofilm-NETs synergistic perspective, our observation that high biofilm forming P. aeruginosa (e.g., PAO1△wspF) induces stronger NETs production aligns with the nutritional/structural requirements of their interaction. Supporting this, strong biofilm-formers (e.g., Klebsiella pneumoniae) promote NETs-related inflammation (38), while *P. aeruginosa* biofilms rely on neutrophil-derived eDNA for stability/immune evasion-eDNA also serves as a core link for biofilm-NETs crosstalk, including biofilm adhesion via eDNA-EPS and NETs bacterial trapping via eDNA scaffolds (39, 40). Clinical CF isolates (strong biofilm-formers) and their virulence factors (ExoS, pyocin S2, pyoverdine) induce distinct NETs (41), and biofilm-like aggregates depend on neutrophil-derived eDNA/F-actin (disruptible by DNase) (42). In this study, the biofilm of the PAO1△wspF strain not only has high yield but also exhibits more complex spatial structures and signaling molecule secretion characteristics, giving it a dual advantage in NETs induction, which further verifies the synergistic regulatory relationship of ‘c-di-GMP-biofilm-NETs.’

The core value of this study lies in the first direct association of the c-di-GMP signaling in *P.aeruginosa* with the mechanism of NETs formation in pleural infections, and the identification of key regulatory nodes such as *Cybb*, *Mapk14*, and *Pik3cd*. This discovery not only fills the gap in the mechanism of ‘biofilm-NETs interaction’ and clarifies the role of c-di-GMP as a ‘signal bridge’ therein, but also provides a new direction for the targeted therapy of chronic pleural infections. By regulating c-di-GMP levels (e.g., inhibiting its synthase) or targeting key genes like *Cybb*, it may simultaneously reduce bacterial biofilm formation and tissue damage caused by excessive NETs induction (25, 43). Subsequent studies can further explore the direct regulatory mechanism of c-di-GMP on these key genes (such as whether it binds to promoters through specific transcription factors) and the therapeutic effects of related inhibitors in animal models, so as to provide more sufficient theoretical and experimental basis for clinical translation.

*P. aeruginosa* strains with varying c-di-GMP levels may induce distinct IL-6, TNF-α, and IL-1β responses during pleural infection. IL-6 requires sustained NF-κB activation and is highest in hypo-biofilm PAO1/plac-yhjH, likely due to continuous PAMP-TLR binding that may prolong NF-κB activity. Hyper-biofilm PAO1△wspF’s matrix may shield PAMPs to blunt NF-κB, potentially lowering IL-6 despite high Mapk14/Pik3cd. TNF-α depends on transient NF-κB activation (44) and peaks in intermediate-biofilm PAO1, which may balance PAMP release and biofilm vesicle supply for optimal signaling. PAO1/plac-yhjH may deplete PAMPs rapidly while PAO1△wspF may block PAMP-TLR interaction, both potentially reducing TNF-α. IL-1β needs NF-κB-driven pro-IL-1β and ROS-dependent NLRP3 inflammasome activation, and is lowest in PAO1△wspF. Its hyper-biofilm may trap ROS to disrupt inflammasome assembly, even with high ROS-generating *Cybb*. PAO1 and PAO1/plac-yhjH have balanced biofilm permeability, potentially enabling ROS diffusion and inflammasome function to support normal IL-1β. A limitation is unvalidated direct PAMP-TLR interaction in biofilm-associated infection, warranting further study.

This study preliminarily reveals the association between the c-di-GMP signaling pathway and host NETs formation in *P.aeruginosa* pleural infection, offering new insights into bacterial-host immune interactions. Future research will focus on core mechanistic details: dynamic *in vivo* c-di-GMP regulation, temporal correlations between NETs formation and bacterial signaling, validating biofilm components’ mediating role via DNase I, and elucidating key genes (e.g., *Cybb*) functions through knockout models. These explorations will improve the theoretical framework of bacterial-host signaling crosstalk and inform targeted therapeutic strategies for pleural infection.

## Data Availability

RNA-seq data have been deposited in the NCBI Sequence Read Archive (SRA) under BioProject accession number PRJNA1373356.

## Supplementary material

Supplementary Table 1 Primer sequences in RT-qPCR.

Supplementary Figure 1 Comparison of *in vitro* c-di-GMP content and biofilm biomass among three *P.aeruginosa* strains. **(A)** The expression of pcdrA-gfp in biofilm in the PAO1△wspF, PAO1, PAO1/plac-yhjH group. **(B)** Comparison of c-di-GMP content measurement in the three infection groups. The optical densities of 600 nm (OD600) and green fluorescent protein (GFP) fluorescence (in relative fluorescence units) (emission wavelength 535 nm; excitation wavelength 485 nm) were recorded. **(C)** Biofilm Biomass of PAO1△wspF, PAO1, and PAO1/plac–yhjH determined by crystal violet staining. Results are represented as the mean ± SD. **P < 0.01, ****P < 0.0001.

Supplementary Figure 2 Biofilm analysis of three *Pseudomonas aeruginosa* strains. **(a)** PAO1△wspF biofilm fluorescence 2D imaging; **(b)** Wild - type PAO1 biofilm fluorescence 2D imaging; **(c)** PAO1/plac - yhjH biofilm fluorescence 2D imaging; **(d)** PAO1△wspF biofilm 3D reconstruction; **(e)** PAO1 biofilm 3D reconstruction; **(f)** PAO1/plac-yhjH biofilm 3D reconstruction. Fluorescence labels: green: viable bacteria; red:dead bacteria. Scale bars represent 50μm.

Supplementary Figure 3 Pro-inflammatory cytokines’s expression in murine pleural lavage fluid. **(A)** IL-6 Levels in Pleural Lavage Fluid of Mice 24 h Post-Infection of Different *P. aeruginosa*. **(B)** IL-1β Levels in Pleural Lavage Fluid of Different *P.aeruginosa*. **(C)** TNFαLevels in Pleural Lavage Fluid of Different *P.aeruginosa*.*P < 0.05, **P < 0.01, ***P < 0.001, ****P < 0.0001, ns: no significance.
