# Supplementary material for: Regulation of neutrophil extracellular trap formation by c-di-GMP in Pseudomonas aeruginosa-induced pleural infection
Source: Front Immunol. 2025 Dec 15;16:1610266. doi: 10.3389/fimmu.2025.1610266 (PMC12745426; doi:10.3389/fimmu.2025.1610266)
Supplement: Supplementary Figure 1 — Comparison of in vitro c-di-GMP content and biofilm biomass among three P.aeruginosa strains. (A) The expression of pcdrA-gfp in biofilm in the PAO1△wspF, PAO1, PAO1/plac-yhjH group. (B) Comparison of c-di-GMP content measurement in the three infection groups. The optical densities of 600 nm (OD600) and green fluorescent protein (GFP) fluorescence (in relative fluorescence units) (emission wavelength 535 nm; excitation wavelength 485 nm) were recorded. (C) Biofilm Biomass of PAO1△wspF, PAO1, and PAO1/plac–yhjH determined by crystal violet staining. Results are represented as the mean ± SD. **P < 0.01, ****P < 0.0001. [file Image1.pdf]

Supplementary Material

1 Supplementary Figures and Tables

1.1 Supplementary Figures

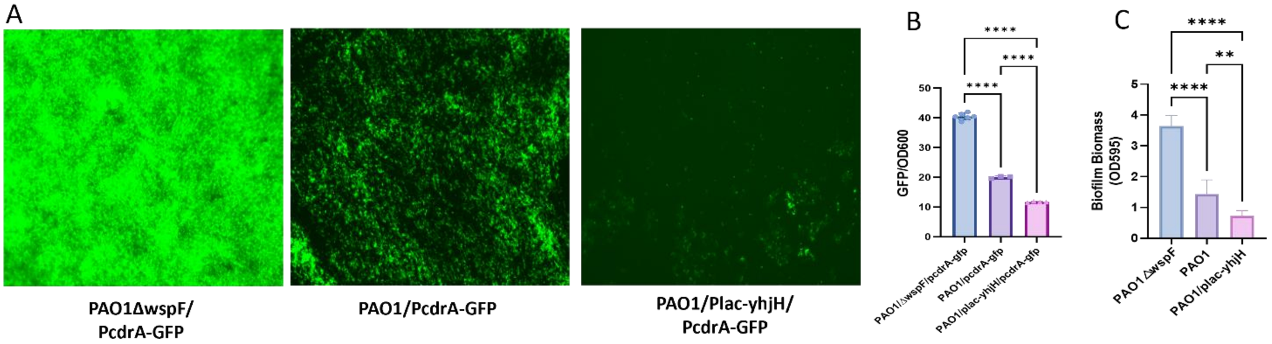

Supplementary Figure 1. Comparison of In Vitro c-di-GMP Content and Biofilm Biomass Among Three *P.aeruginosa* Strains. (A)The expression of pcdrA-gfp in biofilm in the PAO1△wspF, PAO1, PAO1/plac-yhjH group. (B) Comparison of c-di-GMP content measurement in the three infection groups. The optical densities of 600 nm (OD600) and green fluorescent protein (GFP) fluorescence (in relative fluorescence units) (emission wavelength 535 nm; excitation wavelength 485 nm) were recorded. (C) Biofilm Biomass of PAO1△wspF, PAO1, and PAO1/plac–yhjH determined by crystal violet staining. Results are represented as the mean ± SD. **P < 0.01, ****P < 0.0001.

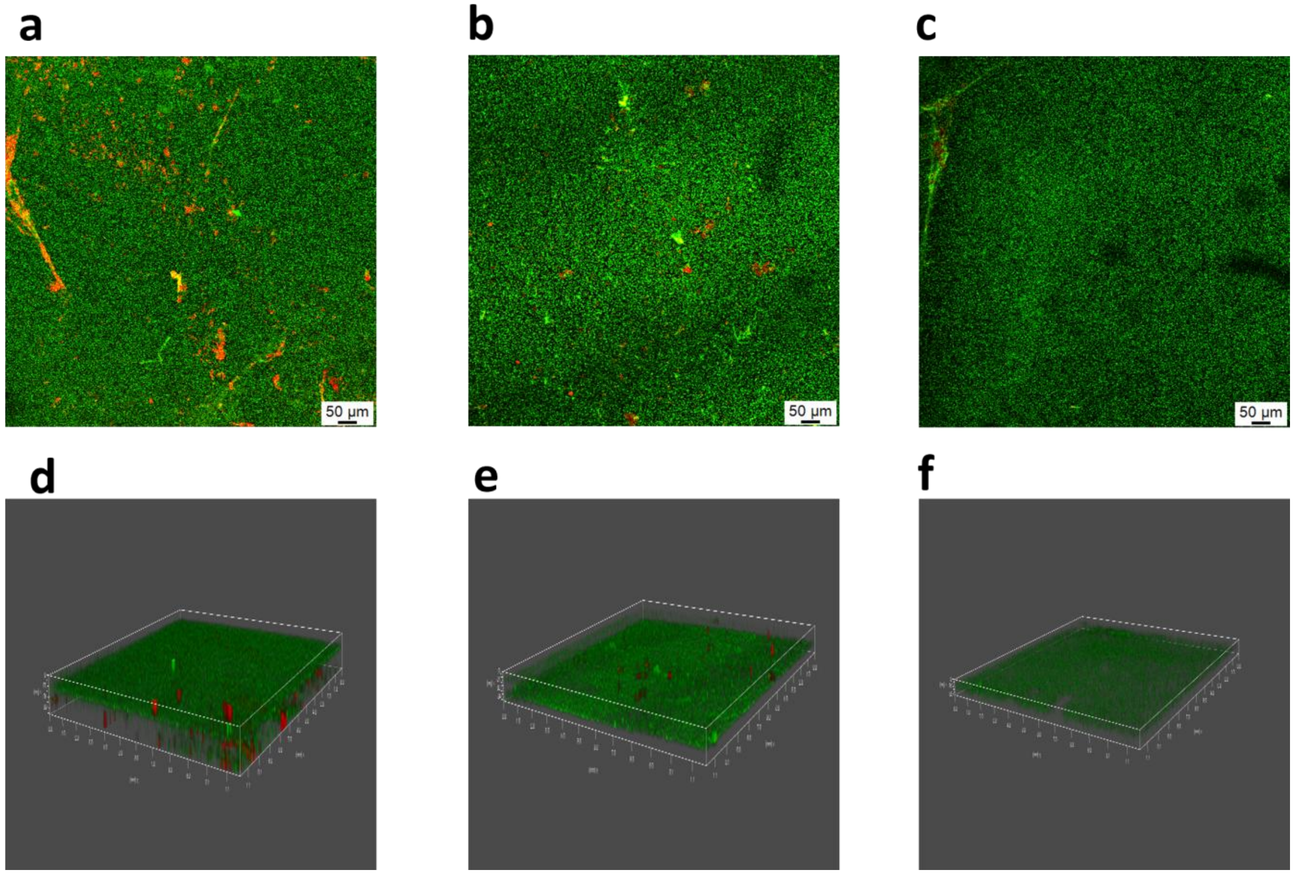

Supplementary Figure 2. Biofilm Analysis of Three *Pseudomonas aeruginosa* Strains.

a: PAO1△wspF biofilm fluorescence 2D imaging; b: Wild - type PAO1 biofilm fluorescence 2D imaging; c: PAO1/plac - yhjH biofilm fluorescence 2D imaging; d: PAO1△wspF biofilm 3D reconstruction; e: PAO1 biofilm 3D reconstruction; f: PAO1/plac-yhjH biofilm 3D reconstruction. Fluorescence labels: green: viable bacteria; red:dead bacteria. Scale bars represent 50 μ m.

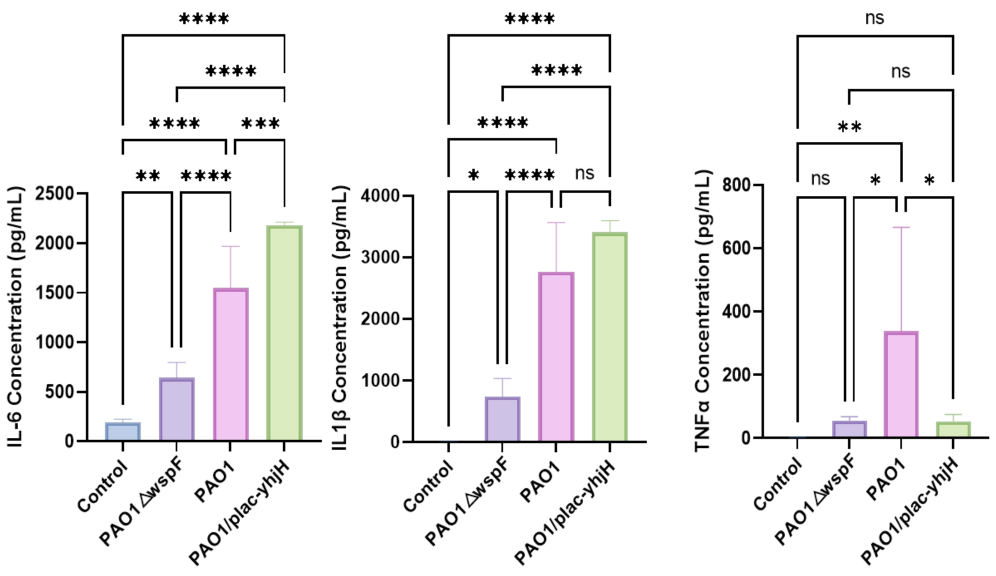

Supplementary Figure 3. Pro-Inflammatory Cytokines's Expression in Murine Pleural Lavage Fluid.

(A): IL-6 Levels in Pleural Lavage Fluid of Mice 24 h Post-Infection of Different *P. aeruginosa*. (B): IL-1β Levels in Pleural Lavage Fluid of Different *P.aeruginosa*. (C): TNF α Levels in Pleural Lavage Fluid of Different *P.aeruginosa*.*$P < 0.05$, **$P < 0.01$, ***$P < 0.001$, ****$P < 0.0001$, ns: no significance.

1.2 Supplementary Tables

Supplementary Table 1. Primer Sequences in RT-qPCR.
